# Beyond mass spectrometry, the next step in proteomics

**DOI:** 10.1126/sciadv.aax8978

**Published:** 2020-01-10

**Authors:** Winston Timp, Gregory Timp

**Affiliations:** 1 Department of Biomedical Engineering, Johns Hopkins University, Baltimore, MD, USA.; 2 Departments of Electrical Engineering and Biological Sciences, University of Notre Dame, Notre Dame, IN, USA.

## Abstract

Proteins can be the root cause of a disease, and they can be used to cure it. The need to identify these critical actors was recognized early (1951) by Sanger; the first biopolymer sequenced was a peptide, insulin. With the advent of scalable, single-molecule DNA sequencing, genomics and transcriptomics have since propelled medicine through improved sensitivity and lower costs, but proteomics has lagged behind. Currently, proteomics relies mainly on mass spectrometry (MS), but instead of truly sequencing, it classifies a protein and typically requires about a billion copies of a protein to do it. Here, we offer a survey that illuminates a few alternatives with the brightest prospects for identifying whole proteins and displacing MS for sequencing them. These alternatives all boast sensitivity superior to MS and promise to be scalable and seem to be adaptable to bioinformatics tools for calling the sequence of amino acids that constitute a protein.

## Teaser

This survey illuminates alternatives for sequencing proteins with the brightest prospects for displacing mass spectrometry.

## INTRODUCTION: CENTRAL DOGMA

The central dogma of biology describes the flow of information encoded in the sequence of nucleotides in DNA into the sequence of amino acid (AA) residues comprising the primary structure of a protein. The information flows first through the transcription of DNA into RNA and then, after processing the RNA into mRNA, by the translation of mRNA into protein. The translation of mRNA into protein is a crucial step toward the ultimate protein structure as the identification of the start site and the open reading frame (ORF) can be problematic (*1*). So, the genetic code, mutations in the coding sequence, and the variability of the start site for translation, along with posttranslational modifications, are all material to the protein sequence, which is the key element that conveys the information about protein structure and chemistry.

Proteins are the molecules that make biology work. They dictate cellular structure and activity, provide the mechanisms for signaling between cells and tissues, and catalyze chemical reactions that support metabolism. The protein structure dictates the function (or dysfunction). Proteins can be the root cause of diseases (such as Alzheimer’s or Huntington’s disease), and they can be used to cure it (e.g., antibodies are used as therapeutics against viral and bacterial infections). It comes as no surprise then that Sanger and Tuppy (*2*) and Edman *et al.* (*3*) analyzed the AA sequences of proteins first, early in the 1950s. Later, teams led by Holley (*4*) working on transfer RNA and Sanger (*5*) working on ribosomal RNA performed the first RNA sequencing. DNA sequencing followed using a variety of methods, both additive and degradative (*6*). Exploiting the development of polymerase chain reaction and other enzymatic methods, DNA sequencing became the focus with relentless improvement in yield, throughput, and cost exceeding “Moore’s law” (*7*), which has been used to gauge improvements in semiconductor device performance over the years. RNA has benefited similarly because of the use of reverse transcriptase to make complementary DNA (cDNA) from RNA, which is then sequenced with DNA sequencing methods. Sequencing DNA and RNA has produced an enormous amount of data. One measure is the size of the sequencing read archive (*8*), a public repository of research sequencing data at the National Center for Biotechnology Information, which currently hosts ~33 P·bases (29 × 10^15^ bases) of data. However, despite the early start, sequencing proteins has lagged behind.

Because they are affordable (the price to sequence a whole genome fell to about $1000 in 2016) and so easy to use, genomic (DNA) and transcriptomic (RNA) sequencing have also been applied to characterize the primary structure of a protein indirectly, but they do not capture the full spectrum of protein-coding genes. For example, paradoxically, an analysis of a “well-characterized” human transcriptome has revealed 116,156 novel transcripts not present in existing databases (*9*). Genome assembly does not perfectly capture protein coding de novo as many assemblies retain an error rate of 0.1%, which, in a 5-Mb genome like *Escherichia coli*, corresponds to about 5000 errors. The insertion/deletion (indel) of a single base, the predominant error in long-read sequencing technologies, can cause a titanic change in the inferred primary structure of the protein by frameshifting. Long-read, human genome assemblies retain indels in as many as 580 assembled transcripts (1.5%) (*10*), which makes it difficult to distinguish mutations from artifacts. On the other hand, frameshifts can be detected easily by looking directly at the AA sequence. Moreover, measurement of RNA transcription offers only a deceptive link to proteins in a cell or tissue—it does not provide a quantitative measure of the protein level (*11*). There are many gene-specific effects in translational efficiency, such as posttranscriptional regulation including RNA modifications (*12*) and even the lengths of the polyA tails added to RNA, that can change the lifetime and the rate of protein production from these mRNAs (*13*), which necessitates unambiguous detection of the protein.

Thus, although it is relatively inexpensive to do so, reading the genome or transcriptome does not buy everything. The prevalence of heterogeneity in mRNA translation (*1*), posttranslational modifications (PTMs) and posttranslational structural processing are revealed only by direct protein-level analysis (*14*), and there is a pressing need for it. However, sequencing a whole protein is a tall order. The primary structure consists of a linear sequence, drawn from 20 proteinogenic AAs with an average volume of about 0.1 nm^3^, linked by peptide bonds separated by only 0.38 nm in equilibrium. Human proteins have about 375 AA residues (*15*). So, several hundred AA calls (acid calls) that discriminate between subcubic nanometer volumes with subnanometer resolution are required to sequence it. Beyond just the 20 proteinogenic AAs, the challenge confronting direct protein sequencing is compounded by isoforms. Isoforms are derived from closely related duplicate genes or the same gene by alternative splicing, proteolytic cleavage, somatic recombination, or PTMs (*16*, *17*). While the number of protein-coding genes is supposed to be about 20,000, taking into account alternative splicing, single-AA polymorphisms, and PTMs, it is estimated that there are north of 100 isoforms per gene (*18*–*26*).What makes protein-level analysis even more daunting are PTMs. PTMs, including glycosylation, methylation, acetylation, and phosphorylation, expand protein chemistry exponentially and affect the structure and function irrevocably, and they are prevalent—e.g., 60% of all proteins are supposedly glycosylated (*26*). Yet, they are difficult to detect by conventional means (*25*).

## BEYOND GENOMICS AND TRANSCRIPTOMICS TOWARD PROTEOMICS WITH MS

Until the early 1990s, protein sequencing was accomplished mainly by Edman degradation. In this process, phenyl isothiocyanate is reacted with an N-terminal amino group to form a phenylthiocarbamoyl derivative that is subsequently cleaved to produce a thiazolinone derivative and a new N terminus. The released thiazolinone AA is stabilized and then identified using electrophoresis or chromatography. This process is then repeated to find the peptide sequence. The Edman process is slow (one cycle takes about 1 hour), and it is limited to peptides less than 30 residues long by the cyclical derivatization, but when done right, it is accurate with >99% efficiency per AA. It requires about 100 pmol of pure peptide, but it does not always report faithfully on PTMs because Edman degradation does not work without a free α-amino group on the N terminus (*27*, *28*).

**Fig. 1:**
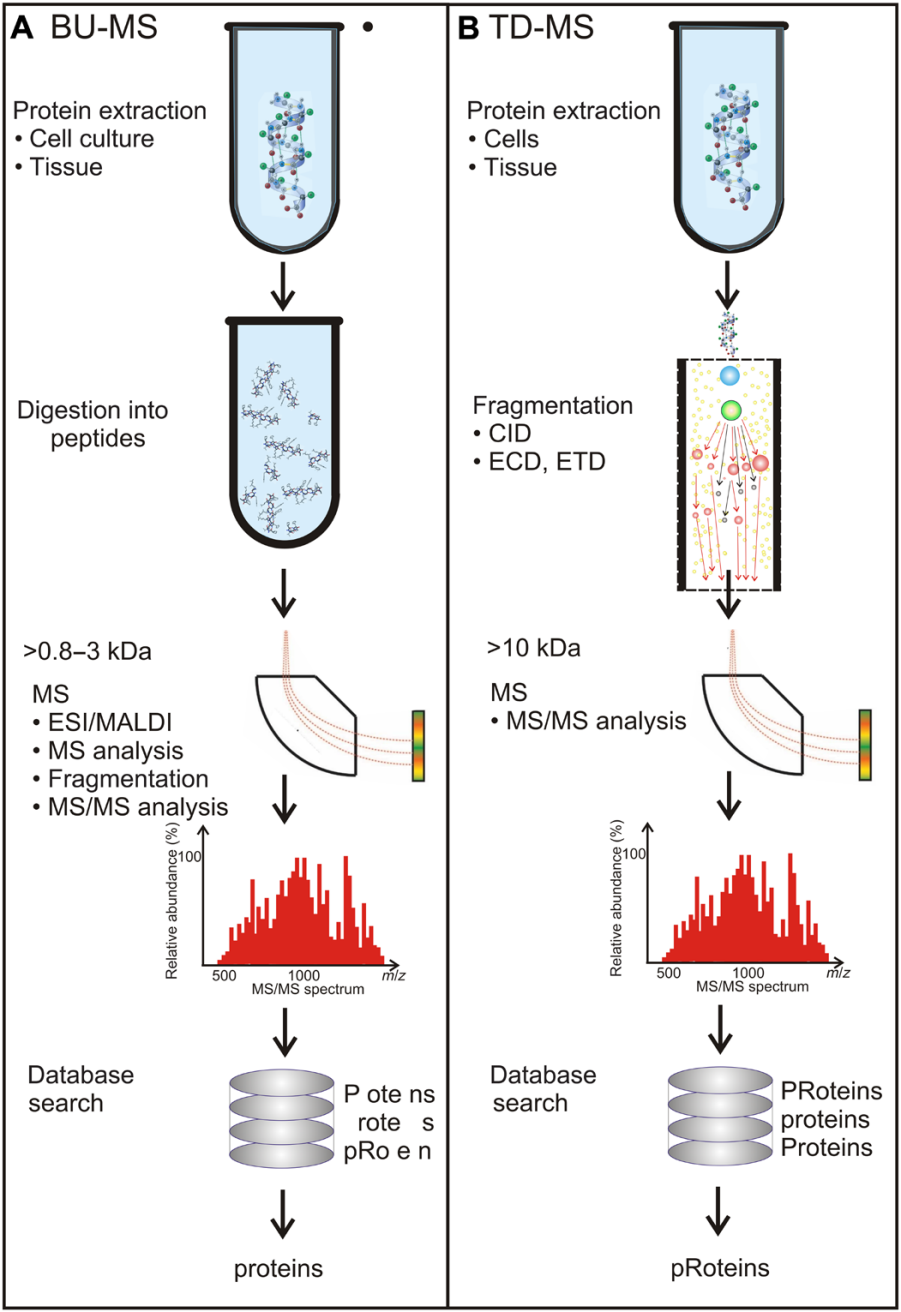
Inferring the primary structure of protein by MS. MS uses two approaches to infer the primary structure of a protein: (**A**) a bottom-up (BU-MS) approach that is used prevalently and (**B**) a top-down (TD-MS) approach that analyzes intact protein <70 kDa. According to the process flow in BU-MS, to infer the primary structure, the proteins are first digested by trypsin, and then the resulting peptides 0.8 to 3 kDa in size, on average, are analyzed in the gas phase by MS. First, the mass of the peptides are determined, and then peptide ions are fragmented to inform on the sequence using tandem MS (MS/MS). BU-MS does not inform on the entire sequence but only fragments as represented by the incomplete word “protein.” In contrast, in TD-MS, intact protein ions are introduced in the gas phase and are fragmented (10 kDa in size on average) and analyzed by MS to identify the mass of the protein and protein ion fragments, which are then puzzled out to reveal the primary structure of the protein. Both methods subsequently rely extensively on the searches through databases to identify the protein. The entire sequence can be revealed this way along with PTMs (represented by the capital letter in the word “pRoteins”). CID, collision-induced dissociation; ECD, electron-capture dissociation; ETD, electron-transfer dissociation.

Currently, proteomics relies mainly on a bottom-up approach to mass spectrometry (BU-MS) for protein-level analysis (Fig. 1A) (*29*–*31*). BU-MS analysis involves enzymatic digestion of the proteins (usually with the protease trypsin), the ionization of the resulting peptides, the separation of the ions according to their mass/charge ratio (*m*/*z*), and then ion detection. The “tryptic peptides” are analyzed by electrospray ionization or matrix-assisted laser desorption/ionization (MALDI) that ionizes peptides in a gas phase, analyzes their masses, and then fragments the ions to recover information about their sequence from MS (*31*, *32*). Alternatively, liquid chromatography–MS (LC-MS) can be used to separate compounds before they are ionized and conveyed into the mass spectrometer.

BU-MS actually does not sequence protein per se but rather infers the primary structure or classifies the protein, and it is not very sensitive (*31*–*35*). The empirical peptide masses act like “fingerprints” that are subsequently correlated with known proteins in databases using search engines such as Mascot or Sequest. Because some AAs have identical masses (e.g., leucine and isoleucine) or nearly so, sequence homology searches are used in conjunction with a database search for sequencing (*33*). One drawback of BU-MS is that the proteins have to be digested into peptides 5 to 20 AAs long before identification. Subsequently, the database searches match only fragments to entire proteins—a process that is frustrated by the sequence homology or similarity shared by the proteins. Last, the assignment of the peptide sequence to a particular protein is accomplished either by inclusion of the peptide with all possible related proteins or by exclusion, which removes any shared peptides before reconstruction, or by parsimony that ferrets out a minimal set of proteins that explains all the observations. These results are then ranked by scores assigned through various methods that compare empirical spectra to theory (*33*).

BU-MS sensitivity is compromised by the number of spectra required to identify the peptide sequences accurately. According to Gris *et al.* (*35*), 75% of the spectra collected remain unidentified because of low signal-to-noise events, the incompleteness of existing databases, and unexpected PTMs. Gris was able to rescue about 20% of the unidentified spectra with clustering, but still, 60% were unresolved and did not inform on the protein. Thus, BU-MS does not inform on the complete sequence but rather identifies a limited number of fragments. Like genomics, the throughput, accuracy, and reproducibility achieved so far with MS have been remarkable (*36*, *37*), but unlike genomics, MS does not offer enough sensitivity and long read lengths.

Sensitivity is paramount. A typical mass spectrometer detection limit is about 480 fg (20 counts/fg), which corresponds to about 10 amol or 6 million, 50-kDa protein molecules (*38*). [Fifty kilodaltons is the median molecular weight (MW) in the human proteome (*15*).] This lack of sensitivity translates to a limited dynamic range. The dynamic range is a gauge of the signal available for detecting peptides or proteins. High dynamic range translates to detection of less abundant peptides in a milieu of more abundant ones. A commercial hybrid Orbitrap used for MS has a dynamic range over about 5 orders of magnitude (Thermo Fisher Scientific), whereas protein concentrations can span 12 orders of magnitude in a clinical specimen. For example, in human serum, antibodies are found at concentrations of milligrams per milliliter, whereas cytokines are found at concentrations of picograms per milliliter (*20*, *39*). Low-MW cytokines secreted in extracellular fluids are prime targets as serum biomarkers, but because of their high biological activity, the concentration is so dilute (low picomolar) that they are practically undetectable by MS without fractionation and enrichment, especially within a background milieu that is human serum (*40*–*45*). Less than 1% of all ions are actually used in the analysis of the mass; however, methods like “Boxcar” that segment the sample by the *m*/*z* improve this fraction and so the dynamic range increases about 10-fold (*46*). Although it has become less reliant on them (*47*, *48*), MS in tandem with assays like multiple reaction monitoring and antibody-based enrichment offer 10,000- to 100,000-fold enhancement in sensitivity with a priori knowledge of the target (*43*, *44*), but still, identification usually requires between about an attomole (a million copies) to about a femtomole (a billion copies) of a protein (*31*, *32*).

Even if the peptide is correctly identified, the search and discovery of isoforms, in general, and the detection and site assignments of PTMs, in particular, are still vexing. MS has been used to ferret out PTMs through enrichment strategies such as ion exchange, immobilized affinity, and chromatography, but they are difficult to execute (*43*, *49*–*51*). Pitfalls include inaccurate mass determination, confusion with residue substitutions, and uncertainty in the site assignment (*49*–*52*). According to Liu *et al.* (*49*), increasing mass measurement accuracy (MMA) whittles down the number of possible AA constituents in a peptide markedly. For example, for identification with high confidence, an MMA of 1 part per million (ppm) can exclude 99% of peptides that have the same nominal mass but different AA constituencies. However, a linear ion trap MS has an MMA of 100 to 250 ppm (*50*); thus, a fraction of proteins can be misidentified. On the other hand, the Orbitrap, which is a workhorse for MS, has an MMA <10 ppm according to the manufacturer’s specification, but to interpret Orbitrap data, up to 50 ppm has been used as the mass tolerance (*51*). Beyond MMA, the uncertainty in the site assignment is especially problematic. Following Kim *et al.* (*52*), the problem can be illustrated succinctly by considering phosphorylation, an important modification for signal transduction pathways that occurs primarily at serine, threonine, and tyrosine residues. There are about 20 million residues in the human proteome (*53*), and the numbers of serine, threonine, and tyrosine residues are about 1.5, 1, and 0.5 million, respectively. So, for a tryptic peptide about 10 AAs long, there are about 1.5 sites for phosphorylation. In other words, there are multiple possible locations of phosphorylation within the peptide. So, the site is assigned statistically, but then, site localization of a PTM is problematic for about half the peptides, or else, a priori knowledge of a PTM is required with BU-MS. This might be addressed by alternative dissociation techniques but then requires more samples and precludes clear examination of combinatorial patterns of the modifications.

On the other hand, top-down MS (TD-MS) identifies intact proteins and can detect sequence variants or provide a scaffold for sequencing, but it is about 100-fold less sensitive than BU-MS; it requires large (>7 to 14 T) magnets and generally lags BU-MS in terms of proteomic coverage and throughput (Fig. 1B) (*31*, *54*–*60*).TD-MS analysis introduces intact protein ions into the gas phase by electrospray ionization that are subsequently fragmented commonly by collision-induced dissociation or, more gingerly, by electron-capture dissociation or electron-transfer dissociation in the mass spectrometer, yielding the masses of both the protein and the fragment ions. With enough fragments, this analysis can provide a comprehensive picture of the primary structure of the protein with its accompanying modifications. However, it is difficult to produce gas-phase fragmentation of intact protein ions for proteins larger than 50 to 70 kDa. It then takes a relatively high-end instrument to resolve the differences between large molecules of similar size. Think about this: The mass difference between lysine trimethylation and acetylation is just 0.0364 Da (*56*). For an average human protein of about 50 kDa, the instrument would have to resolve <1 ppm for an intact protein ion. However, for a 1-kDa fragment, the resolution required is only <37 ppm. A linear quadrupole ion trap/Fourier transform ion cyclotron resonance mass spectrometer with a 7-T magnet has a mass accuracy of only 2 ppm, whereas <10 ppm is typical using collision-induced dissociation fragmentation with an Orbitrap (*49*–*52*, *55*).

According to Steen and Mann (*60*), the sensitivity and detection limit of MS for proteins is just much poorer than that for peptides. As the MW increases, the fragmentation efficiency of intact proteins degrades because of the complexity of the tertiary structure. So, heavy MW then requires high protein purity and high concentrations (0.5 to 1 μg/ml). Thus, most of the top-down applications focus on proteins <70 kDa, with only a few working on larger proteins (>100 kDa) (*54*, *61*).

So, what is needed for directly identifying whole proteins that span the human proteome is a tool that is affordable and accurate enough with extreme sensitivity and high throughput. Ideally, the tool would “read” the AA sequence, PTMs and all, to reveal the primary structure of an isoform directly without appealing to searches through a database.

## THE FIRST TENTATIVE STEPS BEYOND MS

There is no shortage of reviews considering how MS can be applied to proteomics—it is a well-trodden path, and the advantages and disadvantages have already been weighed (*29*–*31*, *33*, *43*, *44*, *52*, *55*–*58*). Here, we offer a less than comprehensive survey (*62*–*64*) of five alternatives with the brightest prospects to displace MS in identifying whole proteins and sequencing them. This review starts with schemes that repurpose state-of-the-art, high-throughput, long-read DNA sequencing for transcriptomics to analyze the true mRNA before translation, including the possible isoforms (*65*–*69*). It is a small step from there to cellular indexing of transcriptomes and epitopes by sequencing (CITE-seq) (*70*), which uses oligonucleotide-labeled antibodies to integrate protein and transcriptome measurements together for an efficient readout. Separate from methods that leverage DNA/RNA sequencing, two strategies are scrutinized for fluorescent protein “fingerprinting” that use a few specific fluorescently labeled residues along with fluorescence microscopy to identify peptide fragments from proteins after they are subjected to consecutive rounds of degradation (*71*–*73*). The sparse fluorescent sequence acquired this way is assigned to a specific protein by alignment to a reference database, which is similar to the workflow used in BU-MS.

The problem with sensitivity hobbling MS can be resolved using a tool of a different stripe, i.e., a nanometer-diameter pore or nanopore through a thin membrane (*74*–*78*). A nanopore is the ultimate analytical tool with extreme single-molecule sensitivity derived from its minuscule volume. It works because the electrolytic current through an open pore immersed in electrolyte changes when a molecule translocates through it, producing a signature blockade current. If the pore is small enough and the membrane is thin enough (and the concentration is dilute enough), single molecules can be detected this way. Commercial platforms that use arrays of nanopores to sequence DNA/RNA are available, so now, we propose to appropriate that same idea for identifying protein.

An elaboration on this theme has been used to extend the protein fingerprinting concept into five dimensions (5D). “5D fingerprinting” of a single native (folded) protein is accomplished by inferring the shape, volume, charge, rotational diffusion coefficient, and dipole moment of a protein from measurements of the modulation observed in the blockade current when it is forced through a pore with a zeptoliter sensing volume (*74*). However, for sequencing, the protein has to be unfolded. Proteinaceous nanopores have been used to discriminate denatured (unfolded) peptides and even identify single-AA differences and PTMs, but not for sequencing protein directly, likely because the sensing volume was just too big. This assertion is borne out by molecular dynamics (MD) simulations that track the translocation of unfolded peptides through a 2.2-nm-diameter pore in atomically thin, 2D materials to reveal how the blockade current coincides with specific AAs in the pore (*79*–*81*). However, the “jamming” of AAs in the pore checks the possibility of determining the AA sequence. On the other hand, using a subnanometer-diameter pore, i.e., a subnanopore, through a thin membrane with a yoctoliter sensing volume, the size of the single-AA residues comprising a single denatured protein can be directly read by measuring the blockade current when the molecule is stretched and forced through it (*75*–*77*).

### Transcriptome sequencing

Second-generation DNA sequencing technology revolutionized genomics by markedly increasing throughput while at the same time reducing the cost, but it uses only short reads (<300 bases), which imposes restrictions on the information gleaned from it. Leveraging this technology, RNA transcribed from the genome and spliced to form the coding sequence has been analyzed to infer the protein primary structure. After transcription, RNA is spliced to remove intronic sequences that do not code for protein and connect the exons that do code for protein at splice junctions usually at a GU-AG site (*82*). Splicing does not just result in different sections of the protein being present or absent; splicing isoforms can result in alternative translation frames, encoding completely different AA sequences from the same genomic locus. When proceeding from the translational start site (usually AUG) through an ORF, every three RNA bases that constitute a codon are translated into an AA. If the reading frame is shifted because it starts in a different place due to the splicing, even the same exon could be translated differently (*1*). Alternative splicing of genes is an active area of study (*9*). Neural tissue, in particular, shows an increased prevalence of longer isoforms that are subject to regulation by neural-specific RNA binding proteins and microRNAs (*83*, *84*).

Although it has improved our understanding of transcript diversity, short-read sequencing informs only on short fragments of cDNA between 50 and 300 base pairs long, and it generally relies on reverse transcription and polymerase chain reaction amplification of the cDNA before sequencing, which introduces bias and is error prone. Therefore, the best that can be hoped for from short-read transcript sequencing is the detection of novel exon boundaries via reads that align across these boundaries and positions of unique start and end sites. To reconstruct the original sequence, short-read sequences are reassembled through alignment algorithms into a full transcript with tools like HISAT2 and cufflinks2 and splicing patterns assessed with tools like leafcutter, MAJIQ, whippet, or SUPPA2 (*85*–*90*). Whereas these studies suggest the potential functional relevance of alternative splicing and polyadenylation sites, they are limited by short sequence reads that cannot always reconstruct the true original sequence and faithfully resolve the complete sequences for transcripts with multiple isoforms. Moreover, direct sequencing of RNA modifications is also precluded, although methods have been developed to indirectly access this information (*91*).

On the other hand, next-generation, long-read (10 kb) sequencing offers more of a direct link that elucidates isoform combinatorics. Pacific Biosystems (PacBio) was the first long-read technology to demonstrate a transcriptome application (isoform sequencing), essentially sequencing entire cDNAs allowing for full isoform generation (*68*). This method has identified more than 12,000 novel isoforms in the GM12878 cell line, which were subsequently validated using short-read sequencing data. Circular consensus sequencing reads were a boon to this analysis, allowing for >99% read accuracy after consensus and read polishing. Powerful software packages, including Cogent, allow clustering and elimination of redundant transcripts and produce a unique set of gene isoforms without the use of a reference (*65*). Long-read transcriptome methodologies have even been used on single cells, a critical application in diverse tissues like the brain (*92*). However, until recently, long-read sequencing has been of limited depth and impractical for determining low-abundance transcripts or work on more rare tissue samples. For example, our ruby-throated hummingbird (*Archilochus colubris*) transcriptome comprised 40 long-read single-molecule real-time flow cells, a substantial expense of both time and treasure (*65*). This has been largely offset by recent marked improvements in long-read sequencing yield, by both PacBio and Oxford Nanopore Technologies (ONT). Another shortcoming is that reverse transcription to DNA is required. Most commercial reverse transcriptases are of limited processivity, which precludes synthesis of especially long or complex structured mRNA (*93*). However, recent developments in nanopore sequencing have allowed for direct sequencing of RNA molecules (*94*–*96*). In particular, long-read sequencing with a nanopore allows for easy resolution of the transcripts without reconstruction; our work (*65*, *66*) and others (*68*, *92*) suggest that there are many unannotated isoforms even in organisms that have been thoroughly analyzed.

**Fig. 2:**
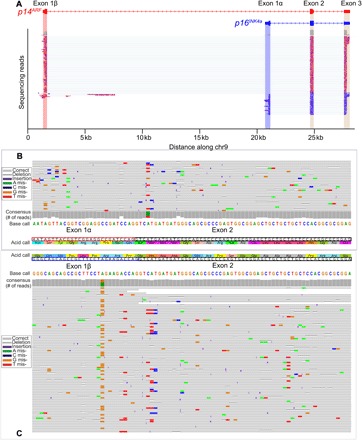
Long-read transcriptomics. (**A**) Direct RNA sequencing reads from GM12878 are shown mapped to the CDKN2B genomic locus. Plotted by Integrated Genomics Viewer, individual RNA reads aligned against the reference human genome (hg38) in this region are shown with the reads matching to the *p14^ARF^* isoform colored red and the *p16^INK4a^* isoform colored blue. Note that exons 2 and 3 are in common, while exon 1α is specific to *p16* and exon 1β is specific to p14. (**B** and **C**) Zoomed-in regions are shown with predicted translations at the 5′ exon 2 boundary. Reads are aligned against the transcriptome (Gencode v27). Integrative Genomics Viewer reads aligned against the transcriptome (Gencode v27) are shown with *p16* on the top and *p14* on the bottom. Although insertions/deletions (indels) and mismatches with the reference are observed, the consensus agrees 99% of the time with the reference. The translated codons are shown between the aligned reads; note that although exon 2 is the same RNA sequence in both, the resulting protein is completely different because of the shifted reading frame from the splice variation.

As an illustration, we specifically examined the *INF4a/ARF* locus using long-read nanopore sequencing data (Fig. 2) (*65*). This locus was chosen because of the interesting frameshift and biological relevance (*97*). This location is transcribed to two primary isoforms in our dataset: *p16^INK4a^* and *p14^ARF^*. First of all, indels and read errors are evident (e.g., Fig. 2, B and C), but after alignment and forming a consensus (Fig. 2, B and C), the read accuracy relative to the reference for this gene improved to about 99%. Although transcribed from the same locus, two of the three exons produce grossly different proteins that act as tumor suppressors for different pathways: RB and p53. Using direct RNA sequencing data obtained from the GM12878 cell line (*65*), we found 93 reads mapping to isoform *p14^ARF^* (Fig. 2A, red) and 33 reads mapping to isoform *p16^INK4a^* (Fig. 2A, blue). Examining the second exon carefully and looking at the corresponding AA sequences that result from translation of this exon in the different isoforms, the proteins are very different—not homologous at all (Fig. 2, B and C; Exon 2, black boxes). The p14^ARF^ translation of exon 2 starts with a glycine (G), straddling the exon-exon border with a GGT→G, whereas p16^INK4a^ starts with a valine (V), starting right at the exon edge with a GTC→V. The frameshift between frame 3 and frame 2 continues through the entire exon and protein. p14^ARF^ does not even make it all the way through the second exon because of a termination about two-thirds through the exon.

So, to correctly identify what protein results from this transcript de novo without complex computational inference methods, either full-length transcript sequencing is needed using PacBio/ONT or the protein has to be sequenced directly. Moreover, in comparison, if it only covered the second or third exons, short-read sequencing could not even be used to identify which isoform was expressed. For accuracy, short reads would have to map exon 1α or 1β to exon 2 boundaries. However, it is more challenging than just examining splicing because a ribosome “picks” the reading frame from the possible reading frames in the transcript. The canonical start site may not always be recognized, and after translating a short ORF, a ribosome can reinitiate translation at a downstream start site. Recent work has demonstrated that frameshifted peptides can be generated from the same transcript (*1*), which underscores the need for protein sequencing above and beyond RNA sequencing.

### Cellular indexing of transcriptomes and epitopes by sequencing

Profiling protein in single cells is difficult, especially for MS because of the amount of material required (*98*, *99*), yet it is critical for dissecting heterogeneous tissue, detecting rare cell states, tracking development, and identifying phenotypes (*69*). Single-cell RNA sequencing has recently emerged to fill this gap with droplet- or well-based cell isolation for extracting and barcoding the RNA (*100*). Despite the volume of data derived from single-cell RNA sequencing—it is estimated that there are about 600 million bases of mRNA in a human cell—computational analysis of the data is still in its infancy, and dropouts associated with incomplete data for individual cells further complicate the issue. These problems are especially relevant to the poor correlation between single-cell protein and RNA levels (*101*).

Protein characterization at the single-cell level usually uses either flow cytometry or fluorescence microscopy. Both rely on immunoreactions (antibodies) to confer specificity and average over multiple cells. In flow cytometry, cells are labeled with fluorescently conjugated antibodies and then passed one at a time through a laser beam to measure the scattered light and fluorescence. Fluorescence microscopy accomplishes the same thing, but instead of the cells moving through a laser beam, the microscope takes images tiled across a slide. Where microscopy has improved resolution of intercellular localization, flow cytometry has superior throughput.

**Fig. 3:**
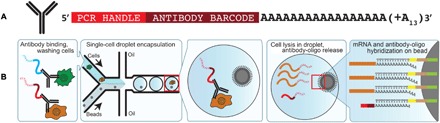
CITE-seq enables simultaneous detection of single-cell transcriptomes and protein markers. (**A**) Illustration of the DNA-barcoded antibodies used in CITE-seq. (**B**) Schematic representation of CITE-seq in combination with Drop-seq. Cells are incubated with antibodies, washed, and passed through a microfluidic chip where a single cell and one bead are occasionally encapsulated in the same droplet. After cell lysis, mRNAs and antibody-oligos anneal to oligos on Drop-seq beads, linking cell barcodes with cellular transcripts and antibody-derived oligos [adapted from Stoeckius *et al.* (*70*)].

CITE-seq combines single-cell droplet RNA sequencing with antibody-based protein profiling (Fig. 3) (*70*). In CITE-seq, cells are first treated with antibodies labeled with DNA barcodes instead of a fluorescent tag (Fig. 3A). These cells are then passed to a microfluidic device that is used to isolate droplets containing (ideally) a single-cell lysis buffer and a microbead covered with barcoded primers as done in droplet sequencing (Drop-seq) (*102*), 10X, or dSeq (Fig. 3B). The cells are lysed, and the RNA and nucleic acid antibody labels are used to generate sequencing libraries with a cell-specific barcode. When these samples are then sequenced, the RNA levels and the amount of antibody present (as a proxy for protein levels) are measured simultaneously per cell. Multiple antibodies can be used and measured per cell; the space of unique DNA barcodes is far larger than the practical abilities of spectral demultiplexing from conventional flow cytometry or fluorescence microscopy. Currently, this practice is limited to cell-surface proteins and is primarily used to link the phenotype of the cells to the single-cell transcriptome. It is possible that CITE-seq could be extended for even greater multiplexing in the future or used for profiling of intercellular proteins in addition to cell surface proteins, giving a clear advantage to characterization (*98*, *99*).

For microscopy, the rapidly expanding field of spatial transcriptomics allows similar simultaneous profiling of RNA and protein. Techniques, such as MERFISH (*103*, *104*), STARmap (*105*), and seqFISH+ (*106*) profile the RNA in individual cells with microscopy using fluorescent probes via either hybridization or even in situ sequencing to identify the RNA population of individual cells. However, the throughput of these techniques is still low, and spectral demultiplexing makes it difficult to combine with immunofluorescent protein staining (*104*).

These methods, along with other protein profiling schemes like enzyme-linked immunosorbent assay and Western blot, all rely on the availability of specific antibodies, but antibodies are expensive and limited by the number of antigens they probe, despite efforts to compile libraries of antibodies (BioLegend’s TotalSeq). Poor specificity can lead to low sensitivity. Whereas the detection limit is supposed to be about 100 to 1000 copies, either false positives from nonspecific binding or false negatives from weak binding make accurate and unbiased measurements difficult. Getting the right conditions for the antibody to bind, avoiding cross-talk between antibodies, making sure that the antigen is available for binding, and even raising the antibody against the correct antigen in the first place all present problems. It is especially hard to analyze the same protein for different PTMs with multiple antibodies due to steric hindrance, i.e., the first antibody will block any subsequent binding. For example, checking to see whether a histone tail has multiple marks (H3K4me3 and H3K27me3) on the same histone requires either sequential immunoprecipitation or other complicated approaches (*107*). So, despite the sensitivity it offers, the limitations inherent to the use of antibodies drive the search for more robust ways to quantify proteins.

### Fluorescent “protein fingerprinting”

**Fig. 4:**
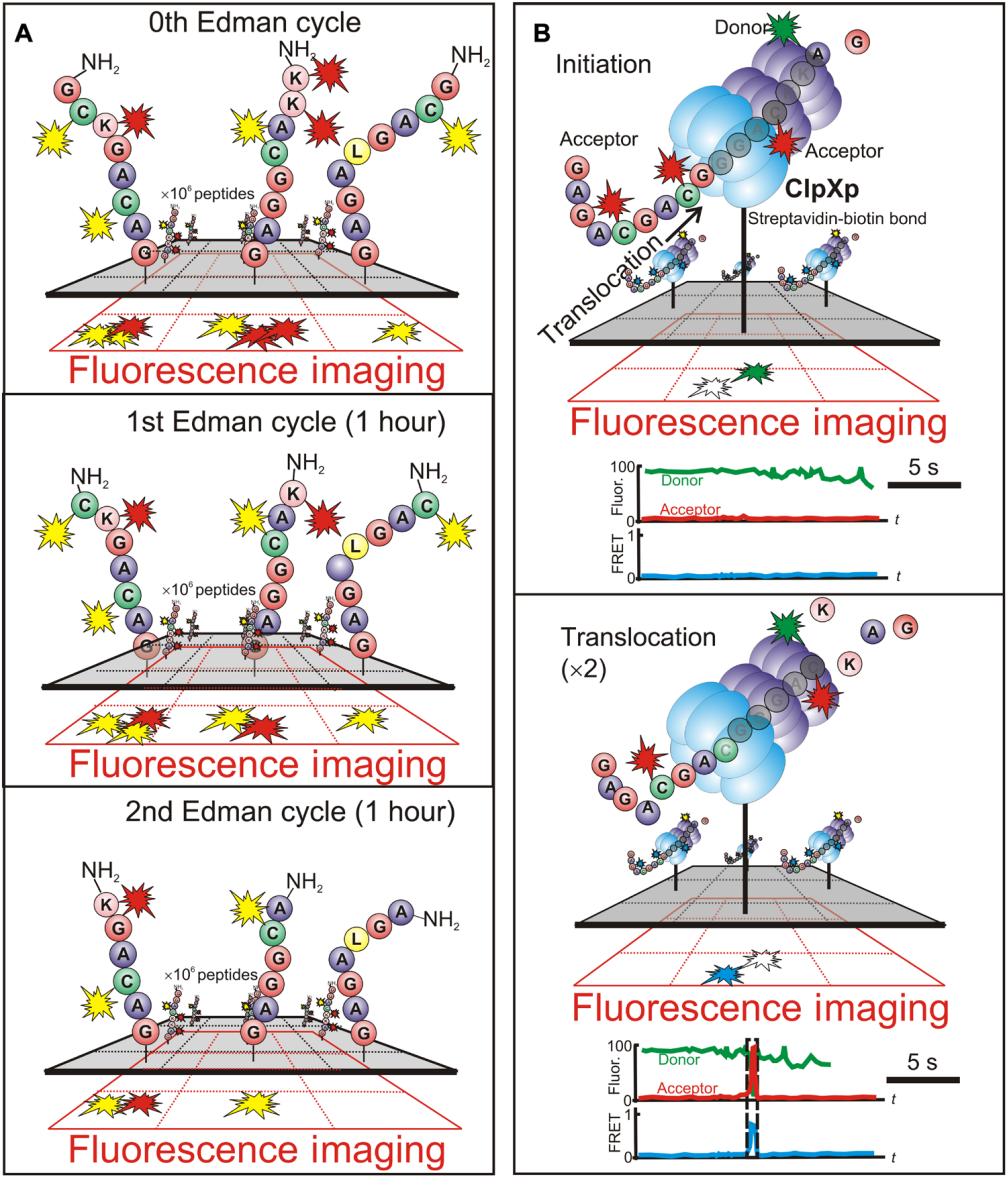
Peptide fingerprinting with fluorosequencing. (**A**) A schematic that represents millions of peptides that are each covalently labeled with (two) different AA-specific fluorescent dyes and immobilized at their C termini using amide linkage to aminosilanes on a glass coverslip mounted on a TIRF microscope stage perfusion chamber [adapted from Swaminathan *et al.* (*71*)]. Through TIRF, each peptide is imaged, and its N-terminal AA is chemically removed via Edman degradation, thus leaving each peptide one AA shorter and regenerating its free N terminus. Repeated cycles of chemistry and fluorescent imaging reveal the positions of fluorescent dyes within each molecule. The pattern of drops in fluorescence intensity is interpreted to provide a partial sequence annotation for each peptide, which can be matched and scored against a protein sequence database to infer the most likely set of proteins present in the sample. (**B**) Schematic of the single-molecule peptide fingerprinting platform leveraging a ClpXP translocation developed by van Ginkel *et al.* (*72*). Donor-labeled ClpXP is immobilized on a polyethylene glycol–coated slide via biotin-streptavidin conjugation. ClpX6 recognizes an acceptor-labeled substrate and translocates it into the ClpP14 chamber, upon which FRET occurs. A typical fluorescence time trace is shown below for each cycle. High FRET reports on the presence of the substrate in ClpP14, whereas the loss of fluorescence signal indicates the release of the substrate [adapted from van Ginkel *et al.* (*72*)].

Protein fingerprinting offers the prospect for identifying (and quantifying) single molecules of protein by tagging specific AAs with fluorescent reporters that can be detected optically. The method is analogous to peptide mass fingerprinting that is used to identify protein fragments by measuring the absolute masses with MALDI–time-of-flight (MALDI-TOF). By marrying the principles used for massive parallel-in-space fluorescence imaging developed for next-generation DNA sequencing with classic Edman degradation chemistry, Swaminathan *et al.* (*71*) have demonstrated a method for fluorescent protein fingerprinting that scales with high throughput for identifying protein fragments by the millions in parallel (Fig. 4A). According to this method, proteins are fragmented and labeled at specific AAs with fluorescent tags. Each fragment is then tethered by its C terminus to a flow cell and imaged with total internal reflection fluorescence (TIRF) microscopy. The intensity of the different fluorescent labels measures the number of a particular label, and hence AAs, present in each fragment. Cycles of Edman degradation are applied, removing a single AA from the N terminus with imaging performed between each cycle. Drops in the fluorescence are then measured versus the degradation cycle to determine the AA position. This method can also be extended to identify the presence and position of PTMs, provided that they can be fluorescently labeled.

An elaboration on this same theme, single-molecule fluorescence resonance energy transfer (FRET)–based “peptide fingerprinting” instead harnesses the AAA+ protease, ClpXP, to scan peptides (Fig. 4B) (*72*, *73*). The ClpXP protein complex is an enzymatic motor that first unfolds the protein using adenosine triphosphate hydrolysis and then degrades it, forcing it to translocate progressively through a central orifice in the molecule. Because ClpXP is so promiscuous toward substrate modifications, including fluorescent labels, donor fluorophore-labeled ClpP can be used to sequentially read out FRET signals from acceptor-labeled AAs constituting the peptide as it is forced through the motor. When the ClpP comes in close proximity to the fluorophore AA, FRET signals the AA type and reveals the peptide’s fingerprint. These signals, in combination with a database search, can be used to infer the identity of the peptide.

Both of these methods are very appealing for protein sequencing because they are scalable and leverage well-worn processes such as Edman chemistry or thoroughly studied proteases that have been engineered for the task, as well as familiar (MS-based) workflows for database searching. Moreover, by using just two labels in conjunction with a database search, even after accounting for errors, it should be possible to infer the identity of the majority of the human proteome.

However, problems remain (*108*). The types of errors generated in these schemes are similar to the errors encountered in DNA/RNA sequencing pipelines, i.e., indels and substitutions. So, as for transcriptome sequencing, the same bioinformatics might be adopted for the error correction. Imagine that each protein read consists of a wild card of AAs at unlabeled positions, anchored by the labeled and known AAs. This protein read can then be aligned against a reference to uniquely identify it. Fluorosequencing by Edman degradation suffers the same problems that Edman degradation has: It is slow, and even at the reported yield per degradation step of >91% (*71*), the length of achievable peptide sequences is limited to <30 AAs by the cycle dephasing. Although specific phosphoserine PTMs have been detected, Edman degradation does not work generally without a free α-amino group on the N terminus. Regardless, the PTMs would have to be specifically fluorescently labeled, but a labeling chemistry is known for only a small subset of PTMs (*71*, *108*). Last, although the methods are scalable up to millions of molecules, the fluorescence still has to be read optically for quantitation across several orders of magnitude in intensity while avoiding photobleaching and dark reads. PacBio and Helicos have hurdled these difficulties with fluorescence readout before. However, as observed by Collins and Aebersold (*108*), the dynamic range of the human proteome (~10^7^), along with the number of peptides per protein produced by enzymatic digestion (~10^2^) and the number of ORFs per cell (~10^4^), still presents a daunting analytical challenge.

### 5D fingerprinting of a folded protein with a nanopore

Like protein fingerprinting, it has been proposed that optical traces obtained from fluorescently labeled protein threaded through a 3- to 5-nm-diameter pore with a plasmonic architecture could be used to identify individual proteins in the human proteome (*109*, *110*), but this scheme suffers the same problems with dynamic range and PTM chemistry. On the other hand, interrogating a whole, single native protein is deceptively simple when it is forced through a nanopore spanning a membrane immersed in electrolyte and the current is measured. When a molecule diffuses up to a pore and is captured by the electric field in it, the ion flow through the pore changes as the molecule translocates through it in proportion to the occluding volume, producing a blockade current that could be measured over a wide dynamic range prospectively (*111*). For pure solutions of protein, a nanopore can be used to detect concentrations ranging from 100 nM to 1 pM, depending on the signal-to-noise ratio. Regardless, structurally similar analytes have been discriminated by the blockade current signature: Low-MW proteins have been differentiated in the secretions from single cells (*112*) and the DNA in a metagenomic (mixture) milieu acquired from (mock) microbial communities (*113*), depending on the acquisition time, the duration of a translocation, the amplifier bandwidth, and the signal-to-noise ratio (*114*).

Naïvely, the fractional change in pore current can be related to the ratio of the molecular volume to the pore volume: i.e., Δ*I*/*I*_0_ = *f* ⋅ Δ*V*_mol_/*V*_pore_ ⋅ *S*, where *f* measures the molecular shape and orientation and *S* is a size factor that accounts for distortions in the electric field that occur when the molecule is comparable in size to the pore (*115*, *116*). To be concrete, the volume of a protein (*V*) scales approximately with MW according to: *V*_mol_(nm^3^) = 1.21 × 10^−3^ × MW(Da) (*117*). So, a (spherical) protein with MW <500 kDa would be about the size of 600 nm^3^ with a radius of about 5 nm, which sets the scale for the pore radius. Using this strategy, a sensitivity of Δ*V*_mol_/*V*_pore_ = 1.2 × 10^−9^ has been achieved detecting 50-nm-diameter nanoparticles in a micropore (*118*), but the sensitivity is generally compromised by noise and biofouling (clogging) as the pore diameter shrinks (*119*). Strategies have been developed to rescue the sensitivity that modulate the blockade current to find signal buried in the noise (*118*–*120*) and preclude biofouling by protein adhering to the pore (*74*, *76*, *112*).

Using the blockade current, nanopores larger than a protein have been used to detect and analyze native or folded proteins before (*121*–*134*). Of particular interest are recent measurements using a wild-type aerolysin nanopore 1 to 1.7 nm in diameter through a lipid layer that have shown that it is possible to discriminate between several short, folded, uniformly charged, homopeptides and even identify single-AA differences (*133*). Likewise, Fragaceatoxin C (FraC) pores through a lipid layer, with a constriction about 1.5 nm in diameter, have been engineered to discriminate peptides and identify single-AA differences, regardless of the charge distribution (*134*). However, it is just not that simple to identify a protein based solely on the blockade current because of limitations imposed by the amplifier and the resulting signal-to-noise ratio. Doubtless, the blockade current is affected by other parameters beside volume such as the protein charge, hydrophobicity, and mobility, and the role these actors play still needs to be assessed to bolster the accuracy as well.

**Fig. 5:**
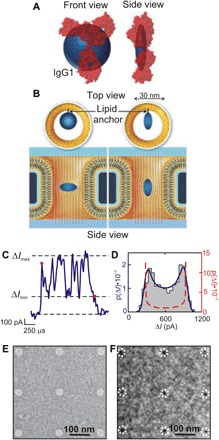
“5D” fingerprinting with a nanopore. Following Yusko *et al.*, the approximate shape, dipole moment, and rotational diffusion coefficient are extracted from current modulations within individual current blockades from the translocation of a single protein through a 30-nm-diameter pore. (**A**) The approximate shape of the antibody immunoglobulin G1 (IgG1) protein as determined by analysis of individual resistive pulses (blue) with crystal structures in red (blue spheroids show the median values of *m* and volume from single event analyses of the protein). (**B**) Top and side views of a 30-nm-diameter nanopore illustrating the two extreme orientations of a spheroidal protein that is anchored to a fluid lipid coating on the pore wall. A crosswise orientation disturbs the field lines inside the pore more than a lengthwise orientation due to the angle-dependent electrical shape factor. Rotational dynamics of individual proteins inside a nanopore reveal a spheroidal approximation of the protein’s shape. (**C**) Current blockade from the translocation of a single IgG1 molecule. Red dots mark the beginning and end of the resistive pulse. (**D**) Distribution of all current values within this one blockade. The dark blue curve shows the solution of the model, *p*(Δ*I*), after a nonlinear least-squares fitting procedure, and the red dashed curve shows the estimated distribution of the blockade current, Δ*I,* values due to the distribution of shape factors, *p*(Δ*I*γ) [adapted from Yusko *et al.* (*74*)]. (**E** and **F**) Arrays of nanopores can be used to boost throughput. A transmission electron micrograph of a nanopore array fabricated using electron beam lithography and reactive-ion etching through a freestanding SiN membrane is shown in (E). The average pore diameter was 29 ± 3 nm [adapted from Verschueren *et al.* (*146*)]. A transmission electron micrograph of a 1.7 × 2.8–μm^2^ silicon nitride membrane 11.3 nm thick with an array of 2-nm-diameter nanopores sputtered on the same 200-nm pitch using STEM is shown.

In a conceptual breakthrough, Yusko *et al.* (*74*) used a nanopore to stretch the idea of protein fingerprinting to unambiguously identify whole proteins by measuring not only the volume but also the shape, charge, rotational diffusion coefficient, and dipole moment this way (Fig. 5, A to D). Using a pore about ~30 nm in diameter through a 275-nm-thick membrane coated with a lipid layer, they analyzed proteins tethered to the lipid layer by tracking how it modulated the blockade current as it translocated slowly across the membrane through the pore. A coarse estimate for the protein volume can be obtained this way (oblate spheroid in Fig. 5A); this has been done before. What is new is the idea that the rotation of a single nonspherical object during a translocation through a cylindrical pore modulates the current blockade (Fig. 5B). The maximum blockade occurs when the spheroidal particle is in its extreme crosswise orientation, whereas a minimum develops in the extreme lengthwise configuration (Fig. 5, C and D). In addition, the protein volume and shape also affect the extent of electric field line distortions, i.e., deviations from a perfect sphere (i.e., oblate spheroid) distort the field lines more markedly. Thus, the magnitude of the current blockade depends on molecular volume and charge, whereas the ratio between the minimal and maximal blockade current modulation depends on the shape. Last, the bias in the distribution of maximum blockade currents is affected by the dipole moment, and the time dependence of the modulation measures the rotational diffusion coefficient. So, the current blockade provides a signature of the protein related to the time-dependent molecular orientation of the molecule in the pore as well as its shape, volume, charge, (rotational) diffusion coefficient, and dipole moment. According to Yusko *et al.* (*74*), measuring these five parameters simultaneously on single proteins in real time can have profound implications for protein analysis: e.g., analyzing proteins one at a time obviates the need for purification.

In principle, an unambiguous “fingerprint” of a protein might be gleaned this way, but timing is everything. To resolve the modulation in the blockade current and recover information about the protein structure, the sampling rate has to be faster than the dynamics of the molecule in the pore. A fast sampling rate also allows for signal averaging that can improve the signal-to-noise ratio. However, because the noise is proportional to the bandwidth, to reduce noise, Yusko *et al.* were forced to narrow the bandwidth of the measurements. Because of the narrow bandwidth and the low sampling rate, to recover information about the structure, the translocation velocity of the protein was slowed commensurately by tethering it to an anchor embedded in the fluid lipid bilayer coating the nanopore, membrane and all (Fig. 5A). In this way, the velocity of the protein through the pore was determined by the 100-fold higher viscosity of the lipid coating instead of the mobility through the aqueous electrolyte in the pore. Without the tether to the lipid layer slowing the molecular velocity, this method falls apart because of the noise and bandwidth. Wider bandwidth usually translates to more noise. On the other hand, the noise could be mitigated, and the bandwidth could be improved if parasitic elements embedded in the electrical network surrounding the nanopore are squeezed out (*135*–*142*).

Even if these problems are resolved, the throughput is too low for practical biology: Only one molecule was detected every 2 s. Borrowing a solution used for sequencing DNA/RNA, arrays of nanopores could be used to rescue throughput. Specifically, the number and density of nanopores through a silicon membrane are inherently scalable with current semiconductor nanofabrication practices. This seems feasible because of the inexorable progress toward nanometer-scale devices that we have witnessed in semiconductor manufacturing. In 2017, IBM working with Samsung and GlobalFoundries announced that it is possible to extend Moore’s law by integrating more than 30 billion gate-all-around transistors with 5-nm gate lengths into a single chip the size of a fingernail (*143*). [Moore’s law (*7*) relates to an observation offered by Gordon Moore in 1965 that the transistor count would double every 18 months to 2 years. Amazingly, semiconductor manufacturing has kept pace with it with unerring accuracy since 1971 using a combination of miniaturization and growing the size of a chip/die.] To cast it in more familiar terms, the A12 iPhone incorporates 6.9 billion, 7-nm gate-length transistors into an 83.3-mm^2^ chip right now (*144*).

Thus, to alleviate the bottleneck in throughput associated with identifying a single protein molecule one at a time with a single pore sampling at a relatively low frequency, it seems feasible to create a dense array of nanopores and measure blockades from them concurrently. The first 30-nm-diameter pore array on a 200-nm pitch has already been carved out of a silicon chip using direct-write electron beam lithography in combination with reactive-ion etching (Fig. 5E) (*145*, *146*). Thinking smaller, arrays of 2-nm-diameter pores on the same pitch can be created too (Fig. 5F), but not with conventional lithography yet. Because the diameter is so small, these were sputtered serially through a thin silicon nitride membrane using a tightly focused, high-energy electron beam in a scanning transmission electron microscope (STEM) (*147*). However, neither of these two schemes for electron beam lithography have proven to be economical for manufacturing.

### Sequencing denatured unfolded protein with a nanopore and subnanopore

**Fig. 6:**
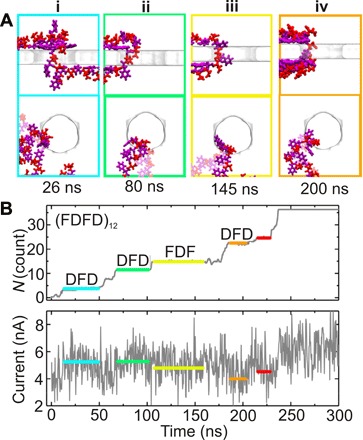
Ionic current blockades produced by an (FDFD)_12_ peptide translocating through a nanopore spanning a 2D MoS_2_ membrane. (**A**) i to iv: Snapshots of the representative conformations of the (FDFD)_12_ peptide translocating through a 2.2-nm-diameter pore at a 600-mV bias at 26, 80, 145, and 200 ns, corresponding to the first, second, third, and fourth translocation pauses indicated by boxes highlighted in cyan, green, yellow, and orange, respectively. The AA phenylalanine (F) is shown in magenta, and aspartic acid (D) is shown in red. (**B**) Top: A tally of the number of AA residues of (FDFD)_12_ peptide that have translocated through the nanopore. The colored horizontal lines highlight the pauses in the translocation. The corresponding AA fragments in the nanopore are indicated above the colored lines. Bottom: In correspondence with (top), the ionic current through the pore is shown. The gray line represents the actual fluctuating current through the pore, while the colored horizontal lines denote the average ionic current at each translocation pause. The color and the length of the line match that from the translocation trace shown in (B) [adapted from Chen *et al.* (*80*)].

Directly reading the primary structure removes the ambiguity in protein identification, but first of all, to access the sequence, the protein has to be unfolded. Five years ago, work by Rosen *et al.* (*148*) and Nivala *et al.* (*149*, *150*) using a proteinaceous nanopore, α-hemolysin, hinted at the prospects. Rosen *et al.* were able to detect phosphorylation of unfolded thioredoxin, and Nivala *et al.* could discriminate distinct domains using ClpXP to unfold a protein, but neither sequenced a protein this way. Nevertheless, it should be possible to read the sequence of an unfolded protein—MD simulations can attest to it (Fig. 6).

By tracking the translocation of artificially denatured peptides and the corresponding blockade current through an idealized model of a pore 2.2 nm in diameter in a membrane made from atomically thin, 2D graphene (*79*) or MoS_2_ (*80*), MD has been used to find several things about sequencing with a nanopore. First, the electric field was focused to a region about 1 nm in extent around the pore waist in a 2D material. This is important because it affects the resolution of a read, i.e., the number of AAs affecting the blockade current and, consequently, the computational gymnastics required to infer the sequence from the blockade current. Second, regardless of the 2D material, the peptide first collapses onto the membrane and is then impelled by an electric force unidirectionally and stepwise through the pore with a velocity that depends on the electric field and/or hydrostatic pressure gradients and water flow. The kinetics of a protein translocating through a pore actually can have two aspects to it: It can either slip-and-stick, unbinding and then binding again to the membrane, or it can slide nearly frictionless through the pore (*76*). According to MD, the stepwise motion yielded fluctuations in the blockade current that informed on the fragment of the peptide in the pore.

Because the entire process was visualized with atomic precision using MD, it was possible to puzzle out just how each residue contributed to the blockade current. In particular, changes were conspicuous between the third pause (yellow, AA fragment in the nanopore, FDF, ionic current = 5.04 nA) and the fourth pause (orange, DFD, 4.29 nA) in the (FDFD)_12_ peptide (Fig. 6B). The same content in the nanopore usually leads to similar currents [e.g., first cyan, DFD, 5.31 nA and second green, DFD, 5.38 nA pauses in the (FDFD)_12_ and second green, FKF, 7.49 nA], but there were exceptions that were attributed to the dependence of ionic current on the conformation of the peptide fragment in the pore. The uncontrolled jamming of AAs in the nanopore destroyed the possibility of determining the AA position based only on measurements of the blockade current—the use of a smaller pore diameter to stretch the protein was indicated (*80*). Whereas MD offered penetrating, atomistic insight into the ion conductance through a nanopore, it was also computationally demanding, and economical simulations of the conductance generally proved to be incommensurate with the limited bandwidth and low electric fields and the narrow electric field distribution (*151*) characteristic of the actual measurements.

So far, proteins have not been sequenced with a nanopore for a few simple reasons. First, a nanopore is likely too big and lacks the sensitivity to discriminate between AAs. If the fractional change in pore current can be related to the ratio of the molecular volume to the pore volume, then a 3-nm-diameter pore with a biconical pore topography in a 10-nm-thick membrane has an effective volume <40 nm^3^, whereas the volume of the smallest AA glycine (Gly) is only about 0.067 nm^3^. On the other hand, a biconical pore with a 20° cone angle and a 0.4-nm diameter has an effective volume <0.6 nm^3^. Thus, in addition to a thin membrane, a much smaller pore volume is indicated for sequencing protein.

A smaller pore diameter also has another advantage: It slows the translocation. According to Muthukumar (*152*), the mobility of a protein in a pore is affected by the excluded volume, the dynamics of the counterions and water there, as well as the interactions with the pore surface. When the pore diameter approaches the hydrodynamic diameter of the protein, the mobility collapses, and the blockade duration increases (1000- to 10,000-fold) (*153*), until, eventually, the molecule fails to permeate through the membrane at all. Thus, a pore of the same size as a protein can be advantageous by increasing the blockade signal measured in the pore volume and slowing the translocation velocity.

Second, to facilitate the interpretation of the blockade current associated with an AA, the protein should be denatured to eliminate the tertiary and secondary structure, leaving only the primary structure. However, the denaturants required to unravel the secondary structure and maintain denaturation are detrimental to proteinaceous pores embedded in a lipid membrane, which are the workhorse platform used to sequence nucleic acids. Thus, the (pore and) membrane has to be mechanically robust and chemically resilient to withstand harsh denaturating agents or high temperature or the high electric fields used to unfold the protein and rehabilitate the pore if it becomes fouled (*154*).

Third, proteins are not charged uniformly, and so, the electric field in a pore cannot provide systematic control of the translocation velocity (*149*). Fluctuations in the velocity could muddle a read. There may be ways around this using unfoldase to unravel a protein into a pore, for example, but the resulting motion through the pore may not be so uniform or even unidirectional (*149*, *150*).

**Fig. 7:**
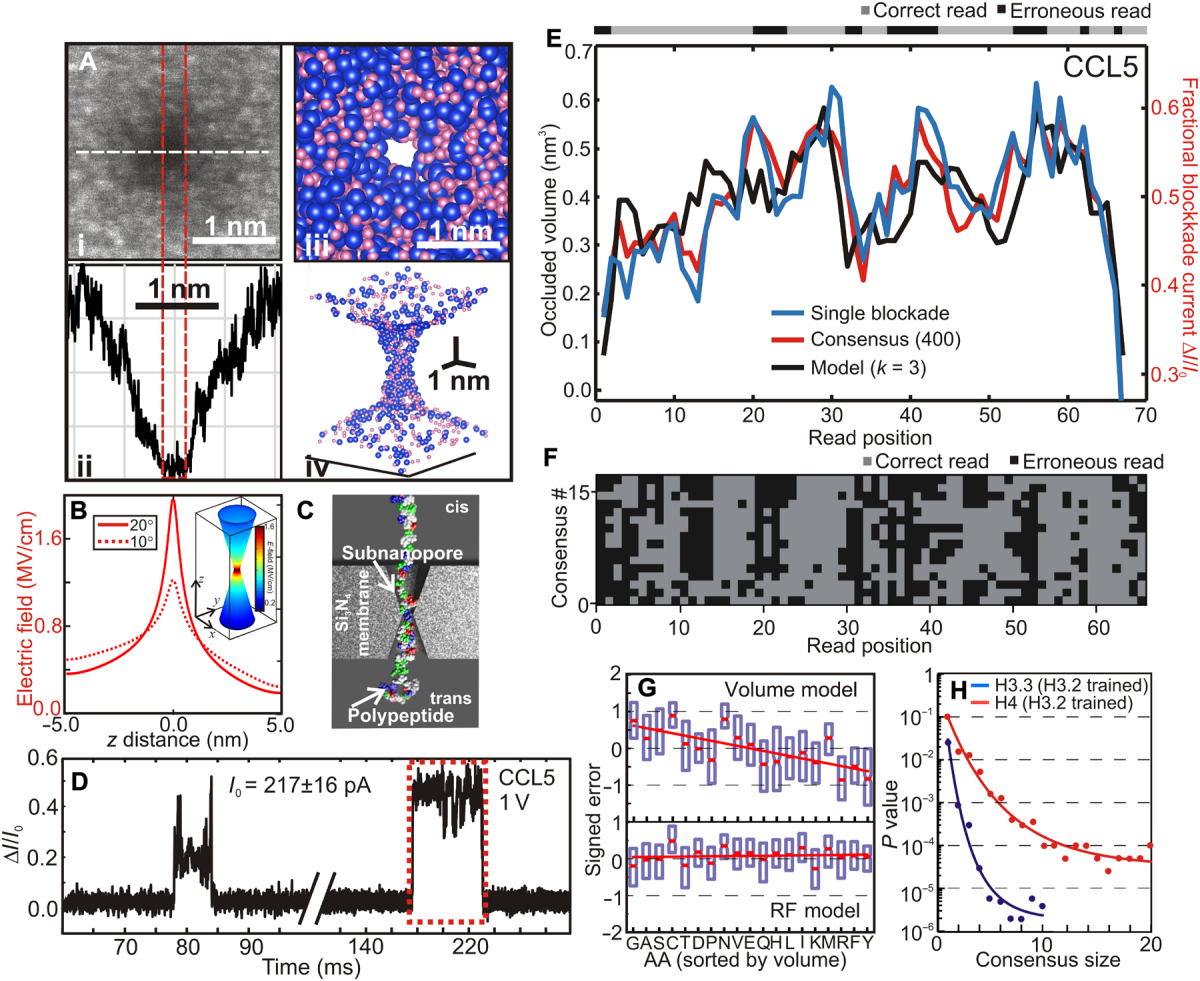
Protein sequence analysis using a subnanopore spanning a silicon nitride membrane. (**A**) (**i**) The topography of a subnanopore is revealed by an HAADF-STEM image acquired with an aberration-corrected microscope. (**ii**) The corresponding line plot through the subnanopore is shown associated with the white dashed line in (i), which indicates the mass density under the beam. The shot noise between the red dashed lines indicates the subnanopore diameter. The subnanopore has a (geometric) mean diameter at the waist of 0.28 nm. (**iii**) A 2D projection from the top through the model that indicates the atomic distribution near the pore waist. The atoms are depicted by space-filling models in which each Si is represented by a blue sphere with a 0.235-nm diameter and each *N* is a pink sphere with a 0.130-nm diameter. (**iv**) A 3D perspective of space-filled representations of the pore model of (iii). For clarity, only atoms on the pore surface are depicted. (**B**) Finite-element simulations of the electric field distribution along the vertical *z* axis of a pore with a 0.4-nm diameter at the waist and a biconical structure with a 10°/20° cone angle through a nominally 10-nm-thick silicon nitride membrane immersed in 250 mM NaCl at 0.6-V bias. The field is focused over an extent of 1.5 nm near the pore waist. Inset: Superimposed on a model of the pore topography is shown a heat map of the field distribution with a 20° cone angle [adapted from Rigo *et al.* (*78*)]. (**C**) A schematic representation of the translocation of a protein through a subnanopore. The denatured protein is supposed to be rod-like. (**D**) Consecutive current traces are shown that illustrate the distribution of the duration and fractional blockade currents associated with translocations of single molecules of CCL5 through a 0.5 × 0.6 nm^2^ pore at 1V. In the figure, higher values correspond to larger blockade currents. (**E**) A 400-blockade consensus (red) for CCL5 through a pore with a 0.5 × 0.6–nm^2^ cross section, juxtaposed with an AA volume model (assuming *k* = 3; black) and a single highly correlated blockade [Pearson correlation coefficient (PCC) = 0.67; blue]. The error map above the plot indicates the read accuracy. (**F**) A grayscale error map of 400 partitioned blockades illustrating correct reads and misreads [adapted from Kennedy *et al.* (*75*)]. (**G**) A comparison between the signed error for AAs constituting H3.2 protein in order of increasing volume naïve volume (top) and random-forest (RF) regression model (bottom) for CCL5. The volume model underestimates signals associated with small volumes, whereas the RF model shows no bias. (**H**) The median *P* value is shown as a function of the number of blockades in a cluster for H4 and H3.3 trained on H3.2. The solid lines represent exponential fits. The decoy database size is 10^5^ for H4 and 5 × 10^6^ for H3.3. The *P* value approaches zero for a consensus >10 [adapted from Kolmogorov *et al.* (*77*)].

It is now technologically within our grasp to fix these problems by creating a subnanopore spanning a thin amorphous inorganic membrane. A subnanopore can be created by sputtering with a tightly focused, high-energy electron beam in a STEM through an amorphous silicon nitride membrane nominally 10 nm thick (*75*–*78*, *147*). Pores made this way generally have a negatively charged surface and can be so small that only single dehydrated cations permeate across the membrane through them (Fig. 7A, i to iv) (*78*). High-angle annular dark-field (HAADF) images acquired with an aberration-corrected STEM (Fig. 7A) have exposed the main structural features of these pores. The HAADF-STEM images acquired under different tilt conditions relative to the axis of the electron beam reveal pores with a biconical topography and an irregular waist >0.25 nm in diameter (*78*). The biconical subnanometer topography is important because it focuses the electric field to a nanometer-scale extent near the waist, and the current density there is proportional to the electric field (Fig. 7B). This means that the membrane does not have to be 2D or single layer of atoms for subnanometer resolution required to see the difference between AA residues spaced 0.38 nm apart (in equilibrium). However, the requirements for fabricating subnanopores outstrip conventional semiconductor manufacturing prowess. Even innocuous processing steps like spinning photosensitive polymer or using remote plasma sources for cleaning the ultrathin membrane before exposure to the tightly focused 60-pm-diameter electron beam used for sputtering a subnanopore in a STEM can compromise yield.

Still, if the thin membrane is small enough in area, it can be mechanically robust and chemically resilient so that it can resist denaturants like SDS, β-mercaptoethanol (BME), acids (HCl), high electric field, and high temperature. Accordingly, proteins denatured by heat, SDS, and BME can be analyzed intact with a subnanopore (*75*–*77*), and a pore that is fouled can be rehabilitated with denaturing agents, although the subnanopore topography through a silicon nitride membrane might etch a bit in HCl (*78*, *112*). Another side benefit is that the SDS used to maintain denaturation of a protein is supposed to form a uniform, negatively charged shell along the protein backbone (*155*) that results in a rod-like molecular configuration (Fig. 7C) and makes systematic electrical control of a translocation possible (although the SDS peels off when the molecule transits the pore waist) (*76*).

When protein denatured with SDS is introduced on the cis side of a subnanopore about 0.5 nm in diameter at the waist and a voltage (<1 V) is applied, short-duration (Δ*t*) blockades (Δ*I*) are observed in the open pore current (*I*_0_) (Fig. 7D) that are attributed to the translocation of single protein molecules. These blockades can be classified by the fractional change in the current (Δ*I/I*_0_) and the duration (Δ*t*). Figure 7 (D and E) illustrates blockades attributed to single molecules of denatured chemokine, CCL5 consisting of 67 AAs. The fluctuations were attributed to a tightly choreographed, turnstile motion of AAs through the subnanopore in which a single AA stalled in a well-defined conformation and then eventually progressed through the pore due to the electric force on the molecule.

The fluctuation amplitudes were correlated with the AA volumes in the primary structure of the protein (Fig. 7E, black trace) (*75*–*77*). Because of the biconical topography, the electric field was focused within 1.5 nm of the waist of a pore. Because the current density was proportional to the electric field, each fluctuation in a blockade actually measured a moving average of AA volumes with a window size (*k*) spanning from *k* = 3 to 5 AAs, corresponding to the extent of the field. This naïve volume model was well correlated to the empirical consensuses formed from the average of blockades normalized in time with the fractional current zeroed (Fig. 7E, black trace). From the signal derived by averaging over a 400-blockade consensus, we found that the Pearson correlation coefficient (PCC) = 0.75 with a *k* = 3 volume model. The resulting acid call accuracy was 65.2% for a 20% threshold tolerance (*75*). Moreover, the agreement with the model improved as the number of blockades in the consensus increased. For illustration, error maps were produced by partitioning 400 CCL5 blockades into 17 consensuses (Fig. 7E), each of which was compared to a *k* = 3 model. The maps indicated that acid call errors occur consistently at particular spots in the sequence attributed to smaller AA volumes (*75*).

However, if the blockade current level is affected by other parameters besides volume, then an algorithm to decode the reads should also be trained to recognize these differences to rescue accuracy. In a recent pilot study (*77*), machine learning algorithms were used to test the prospects. A random-forest (RF) model was benchmarked in the pilot study. The model was compared with the volume model, which assumed that each fluctuation corresponded to a quadromer read. Initially, each quadromer **q**_i_ from the training set was converted to a feature vector **f**_i_, where each element of the vector consisted of a volume and hydrophilicity. The training sets were expanded by randomly permuting the AAs in each **f**_i_ while maintaining the corresponding **q**_i_. In contrast to the volume model, the RF model was robust to outliers with less overfitting.

The RF model performed well on the training sets and demonstrated significant improvement over the volume model measured by PCC (Fig. 7, G and H) (*77*). Moreover, an error analysis revealed a bias in the signal estimation that was correlated with the AA volume and hydrophilicity. The bias was calculated from the mean difference between empirical and theoretical blockades (Fig. 7G). Whereas the volume model showed a bias with a larger volume/hydrophobicity having a disproportionate effect on the blockade, the RF model showed none. Thus, for improved statistical significance, additional AA features should be incorporated into the model.

Further analysis of the blockades from a subnanopore with the RF model revealed that it was possible to identify one protein in a database covering a small proteome, i.e., 20% of the human proteome, with a cluster of only 5 to 10 blockades with *P* values ranging between 10^−4^ and 10^−6^ (Fig. 7H) (*77*). Likewise, database searches through small proteomes using TD-MS data indicate that reliable identification of a protein can be accomplished with protein-level *P* values of 10^−4^, which are computed by combining individual peptide identifications (*156*, *157*). However, the protein-level *P* values deteriorate from the peptide level accomplished with TD-MS, which can compromise the sensitivity (*156*, *157*).

As a measure of the sensitivity, 1-fmol sensitivity standards are typically included in a MALDI-TOF/TOF run used for protein identification (*158*), which translates to about 600 million molecules. A similar number of molecules (600 million) is likewise found in 100 μl of a pure solution of 10 pM protein analyzed with a subnanopore embedded in a microfluidic device (*159*). This is not a true gauge of the subnanopore sensitivity; instead, it measures the diffusion capacitance that governs the time (1 s) required to capture a single molecule (*135*, *159*). The extraordinary sensitivity of a subnanopore becomes apparent when the diffusion capacitance is eliminated by placing the molecular source in proximity to the pore. For example, it is possible to discriminate a single blockade or one molecule associated with a particular protein in a mixture that constitutes the secretome of a cancer cell when it is placed within 10 μm of a pore to reduce the diffusion capacitance (*112*).

Last, scrutiny of the fluctuations in blockades acquired from 21 different proteins has subsequently revealed that a subnanopore is sensitive enough to read the occluding volumes due to PTMs of a single residue (*75*) or residue substitutions, measuring differences of ~0.07 nm^3^ between just two molecules (*76*). However, a subnanopore is still not sensitive enough to discriminate all the AAs by volume alone.

To improve the sensitivity, the sampling has to be extended to high frequencies to promote signal averaging commensurate with the velocity of an AA residue through a subnanopore, and the corresponding current noise has to be abated. Signal averaging can be accomplished in two ways by (i) oversampling the signal and then filtering it and/or (ii) acquiring multiple ostensibly identical copies with exactly the same stimulus and averaging them. Both schemes benefit if the noise is abated. However, current noise in a pore is inescapable, and mitigating it is the “holy grail” of sequencing. Analyses of the current noise power spectra have revealed four components to the noise (*78*, *135*, *137*, *141*): (i) thermal noise associated with the resistance of the electrolyte and the pore, (ii) 1/*f* noise, (iii) dielectric noise associated with the membrane, and (iv) amplifier noise. Analyzed this way, 1/*f* noise has actually attracted undue attention because the spectra have revealed that the total integrated noise power in the range >1 kHz is dominated by dielectric noise, which is related to the amplifier’s voltage noise acting across the total capacitance at the amplifier input (*135*, *141*). Thus, schemes to mitigate the parasitic dielectric noise associated with the membrane have been a focus for research. These efforts include using a laminated polyimide layer with a small relative permittivity (κ) to reduce the parasitic capacitance (*135*) and sandwiching a membrane in a low-κ dielectric (*136*, *141*, *142*). Another focus has been low-noise, on-chip amplifiers (*138*–*142*).

There is another problem: systematic control of the translocation velocity. The mobility of the AA through a subnanopore is likely affected by its size, hydrophobicity, and charge, and so, the velocity in the electric field will fluctuate, which could scramble a read. ClpXP has been used successfully to unravel a protein into an α-hemolysin pore, but this unfoldase has yet to be coupled to a pore through an inorganic membrane (*149*, *150*). It may not be necessary though. We speculate that the steric hindrance associated with the SDS that adheres, on average, about every two AAs along the protein backbone effectively checks the progress of the SDS-protein agglomerate through a subnanopore, forcing halting steps in the translocation before it peels off (*76*).

## WHAT IS THE NEXT STEP IN PROTEOMICS?

These examples illuminate the salient features of some of the brightest prospects outside MS for sequencing protein. They all boast sensitivity superior to MS achieved using either fluorescent reporters, immunoreactions, or minuscule volumes for detecting AAs. Likewise, it should be possible to scale them all either by tethering millions of molecules to glass or through semiconductor nanofabrication to economically multiply the number of devices used for detection. Adapting the bioinformatics tools used so successfully to align and call nucleotides in a DNA sequence or used in the MS workflow to infer the protein structure, it seems computationally tractable to analyze the sequence of AAs that constitute a protein as well. Imagine being able to quantify the proteome of cells and tissues as easily as we can currently measure the genome or transcriptome today. The explosion of data and the penetrating insight that developed from the original draft of the human genome could be expanded to include the proteome and so produce a comprehensive understanding of cellular processes and how they are regulated. Moreover, expanding the notion of “molecular diagnostics” beyond antibody-based assays would be a boon to clinical medicine.

However, challenges still remain to be overcome. Long-read transcriptomics is error prone, making exact resolution of isoform regulation difficult. It also measures RNA, not protein, which precludes precise quantitation, let alone PTM measurement. Linking proteomics to DNA/RNA sequencing on a single-cell basis (e.g., CITE-seq) is still challenging. Current methods only profile cell surface proteins and may have logistic challenges associated with antibody interference. Fluorescent fingerprinting methods that infer the protein sequence are scalable but are likely to be plagued by problems common to single-molecule fluorescence detection (Helicos), e.g., dark reads. Fluorescent fingerprinting can only probe specific taggable side chains, precluding a comprehensive analysis of PTMs. Nanopore 5D fingerprinting also requires chemistry, but it needs not be so if the pore size is reduced and the protein mobility in the pore collapses. However, as the pore size shrinks, so does the dynamic range. On the other hand, the massive parallelism that rescues techniques, such as CITE-seq and fluorosequencing by Edman degradation, has not been implemented for nanopores yet, although semiconductor manufacturing offers that promise.

On the immediate horizon (right now, near-term), MS, which is not so sensitive and does not inform on the sequence of whole proteins, will likely be augmented by long-read transcriptomics and CITE-seq/spatial transcriptomics by simultaneous profiling of specific protein and nucleic acid signatures. In the near-term, fluorescent fingerprinting methods may occupy a space as soon as in a year or two with instruments that can generate millions of single protein reads for using fluorosequencing by Edman degradation, but it will be slow because each cycle takes an hour. In the longer (5-year) term, once the kinks with throughput, bandwidth, and noise are worked out, it seems likely that arrays of subnanopores for profiling protein will assume center stage, given its extreme sensitivity and prospects for scaling.
